# Deep-Learning-Based Classification of Lung Adenocarcinoma and Squamous Cell Carcinoma Using DNA Methylation Profiles: A Multi-Cohort Validation Study

**DOI:** 10.3390/cancers18040607

**Published:** 2026-02-12

**Authors:** Maram Fahaad Almufareh, Samabia Tehsin, Mamoona Humayun, Sumaira Kausar, Asad Farooq

**Affiliations:** 1 Department of Information Systems, College of Computer and Information Sciences, Jouf University, Sakaka 72388, Saudi Arabia; 2 Center of Excellence—AI, Bahria University, Islamabad 44000, Pakistanasadkhosa330@gmail.com (A.F.); 3 School of Computing, Engineering and the Built Environment, University of Roehampton, London SW15 5PJ, UK

**Keywords:** DNA methylation, lung cancer classification, deep learning, LUAD, LUSC, TCGA, epigenetics, SHAP analysis

## Abstract

**Background/Objectives:** The precise classification of non-small-cell lung cancer (NSCLC) into lung adenocarcinoma (LUAD) and lung squamous cell carcinoma (LUSC) has important role in treatment decisions and in prognosis. Proper subtyping ensures that patients receive the most appropriate therapeutic strategies and allows clinicians to make informed evaluations regarding disease outcomes. This study presents a deep neural-network-based classification approach utilizing genome-wide DNA methylation profiles from the Illumina HumanMethylation450 BeadChip platform. **Methods:** A total of 5000 of the most discriminative CpG probes are identified through variance-based feature selection in the presented methodology, which are then classified through a five-layer deep neural network with batch normalization and dropout regularization. Training and validation were performed using data from The Cancer Genome Atlas (TCGA), with external validation conducted on two independent Gene Expression Omnibus (GEO) datasets: GSE39279 and GSE56044. **Results:** The model achieved 96.92% accuracy with an area under the receiver-operating characteristic curve (AUC-ROC) of 0.9981 on the TCGA test set. Robust generalization was obtained in cross-dataset validation experiments, with the GEO-trained model achieving 88.92% accuracy and 0.9724 AUC-ROC when validated on TCGA data. The most influential CpG biomarkers contributing to classification decisions are analysed using SHAP (Shapley Additive Explanations). **Conclusions:** These findings demonstrate the potential of DNA methylation-based deep learning approaches for reliable NSCLC subtype classification with clinical applicability.

## Simple Summary

Lung cancer stands as the leading global cancer killer which claims more lives than all other types of cancer combined. Doctors need to determine which lung cancer type patients have between adenocarcinoma and squamous cell carcinoma because these cancers need different treatment methods. A patient will not receive suitable treatment options for their condition when their medical condition remains unidentified by mistake. The research team developed a computer system which analyzes DNA methylation patterns to identify between these two cancer types. Our program learned from data on over a thousand patients and correctly identified the cancer type about 97 percent of the time. The system underwent testing on two distinct patient populations to verify its effectiveness across different patient groups rather than limited training data. The explanation tools we applied demonstrated which DNA markers hold the most importance, while showing our entire method in detail. The research gives medical personnel methods to diagnose diseases both quickly and precisely.

## 1. Introduction

Lung cancer remains the leading cause of cancer-related mortality worldwide, accounting for approximately 1.8 million deaths annually [1]. According to the American Cancer Society, an estimated 226,650 new cases of lung cancer and 124,730 deaths are projected in the United States for 2025, representing approximately one in five of all cancer-related deaths [2]. The disease predominantly affects older populations, with the average age at diagnosis being approximately 70 years, and the overall five-year relative survival rate remains at 27% [3].

Non-small-cell lung cancer (NSCLC) constitutes approximately 87% of all lung cancer cases, with the remaining 13% classified as small-cell lung cancer (SCLC) [2]. The two predominant histological subtypes of NSCLC are lung adenocarcinoma (LUAD), accounting for approximately 40% of lung cancers, and lung squamous cell carcinoma (LUSC), representing approximately 25% of cases [4]. Accurate determination of the subtype has great clinical significance, since treatment strategies, their efficiencies, and prognosis also vary significantly among LUAD and LUSC [5]. While most adenocarcinomas develop in the outer areas of the lung and are the most common type among nonsmokers, most squamous cell carcinomas originate in the central airways inside the lungs near the bronchi and are associated with heavy tobacco use [3].

In the last few years, the treatment of non-small-cell lung cancer (NSCLC) has evolved substantially. This progress is primarily attributed to the development and implementation of targeted therapies and immunotherapies. Molecular profiling shows that EGFR mutations and ALK rearrangements are found mainly in adenocarcinomas, which supports the use of tyrosine kinase inhibitors as targeted treatment [6]. In contrast, squamous cell carcinomas show distinct molecular features and may be susceptible to specific treatments. Accurate histological subtyping is not only an academic classification; it is a clinical requirement that directly guides treatment choices and influences patient outcomes.

DNA methylation is an epigenetic change in which a methyl group is added to cytosine bases, most often at cytosine–phosphate–guanine (CpG) dinucleotide sites [7]. Abnormal DNA methylation is a common feature of cancer, and methylation patterns that are specific to tumors can serve as biomarkers for cancer detection, grouping of tumor types, and estimation of clinical outcomes [8]. The Illumina HumanMethylation450 BeadChip measures DNA methylation at more than 450,000 CpG sites across the genome and has been widely used for genome-wide methylation profiling in cancer studies [9].

Applying machine learning and deep learning methods to high-dimensional biological data has produced useful results for cancer classification in research settings. Deep neural networks can learn complex non-linear relationships in data, which allows them to detect subtle patterns that standard analytical methods may miss [10]. Combining DNA methylation data with deep learning models may support the development of reliable cancer classification methods that are suitable for clinical use.

This study proposes and tests a deep learning classification model to distinguish LUAD from LUSC using genome-wide DNA methylation data. This study uses data from The Cancer Genome Atlas (TCGA) to develop the model and tests it on independent datasets from the Gene Expression Omnibus (GEO) for external validation. Interpretable machine learning methods, in particular SHAP (Shapley Additive Explanations), are used to explain which biological features drive the model’s classification decisions, improving transparency and supporting the search for potential methylation biomarkers.

The primary contributions of this research are listed below.

- The establishment of a five-layer deep neural network architecture incorporating batch normalization and dropout regularization for LUAD/LUSC classification using DNA methylation profiles.
- The establishment of a thorough validation framework across multi-cohorts assessing model generalizability within TCGA and two separately obtained datasets from GEO (GSE39279, GSE56044).
- Experiments were conducted on training datasets showing that GEO-trained models significantly outperformed TCGA data in external validation (88.92% accuracy, 0.9724 ROC-AUC).
- The creation of a SHAP-based model explaining the most influential CpG biomarkers driving classification.

The rest of this paper is organized as follows. Section 2 provides a review of the previous research on cancer classification using DNA methylation data, as well as an overview of the machine learning approaches applied in lung cancer studies. Details of the proposed methodology, data processing, model development, and employed evaluation metrics are discussed in Section 3. Section 4 presents the experimental results, including both internal and external validation across multiple datasets, along with an interpretability analysis using SHAP. Section 5 discusses the findings, their relevance to clinical practice, and the limitations of the study. Section 6 concludes the paper by summarizing the main contributions and suggesting directions for future research.

## 2. Literature Review

The use of machine learning for cancer classification using DNA methylation data has obtained good progress in recent years. This section reviews the most significant studies that have employed artificial intelligence, machine learning, and deep learning approaches for lung cancer subtype classification and related cancer tissue-of-origin identification using DNA methylation profiles.

In their 2021 article, Modhukur et al. [11] perform an excellent experiment on the classification of primary and metastatic cancers. In their research, they employ DNA methylation profiles out of the tissue of origin to enhance the precision of cancer classification. The analysis used 9303 samples from the TCGA and GEO databases of methylomes, which includes 24 cancer types, such as LUAD and LUSC. The authors applied various machine learning algorithms in order to carry out their analysis. In particular, they used random forest, support vector machine, XGBoost, and naive Bayes. In order to obtain optimal results in regard to interpretability of these models, they included LIME (Local Interpretable Model-Agnostic Explanations), the results of which provided insight into the decision-making process of each algorithm, and they overcame the problem of class imbalance with the use of SMOTE oversampling.

Zheng and Xu [12] present a deep neural network model to predict the origin of cancer based on DNA methylation data of 7339 cancer types in TCGA (including LUAD and LUSC among 18 cancers). Their DNN model was composed of 10,360 input neurons (in this case, they were selected as CpG probes); two hidden layers, each with 64 neurons; and an output layer with 18 classes, which were trained in TensorFlow. The 10-fold cross-validation showed that the model had a specificity of 99.72%, sensitivity of 92.59% and positive predictive value of 95.03%. External validation was done on 581 GEO samples with 99.91% specificity and 93.43% sensitivity.

Jurmeister et al. [13] refer to the clinically demanding problem of classifying primary lung squamous cell carcinomas (LUSC) versus head and neck squamous cell carcinoma (HNSC) metastases based on DNA methylation profiles. Artificial neural networks, support vector machine and random forest were used in the study, with all of them trained on the data provided by the Illumina 450K methylation array. The artificial neural network classifier was the most accurate among them, with an accuracy rate of 96.4%. The support vector machine achieved a accuracy of 95.7% and the random forest approach achieved 87.8% to validate the robustness of the results. The test was done on an independent cohort of 279 samples. The findings declared the clinical significance and consistency of methylation-based categorization in this regard.

A machine learning process has recently been proposed by Al-Qirshi et al. [14] to determine lung cancer subtypes using DNA methylation data as their input. The paper utilized methylation data available in The Cancer Genome Atlas (TCGA), including data on 311 patients with lung adenocarcinoma (LUAD), and lung squamous cell carcinoma (LUSC). Illumina HumanMethylation27 was used to analyze these samples. The author used both the random forest and CatBoost classification algorithms on the dataset. The random forest model was excellent, and it achieved a 97% classification accuracy for LUAD and LUSC.

Cai et al. [15] proposed DeepMeth, a novel deep methylation representation learning framework for noninvasive lung cancer detection, which was presented at AAAI 2022. The study utilized an autoencoder-based architecture to learn discriminative methylation representations from multicenter clinical data spanning 14 hospitals. The deep learning approach demonstrated substantial improvements of 5–8% in AUC compared to traditional machine learning baselines, highlighting the capacity of deep neural networks to capture complex, non-linear methylation patterns.

Tang et al. [16] developed an integrative approach combining miRNA expression and DNA methylation markers for tumor origin detection. Using machine learning classifiers trained on TCGA data, including lung cancer subtypes, the study demonstrated that DNA methylation features provide complementary information to gene expression for accurate tissue-of-origin classification, with methylation markers contributing significantly to the discrimination of closely related cancer types.

Moran et al. [17] reported a study in *The Lancet Oncology* that examined epigenetic profiling as an approach for classifying cancer of unknown primary (CUP). The EPICUP classifier uses a random forest model trained on DNA methylation profiles from 2790 samples covering 38 tumor types, including lung adenocarcinoma and squamous cell carcinoma. In a validation study using 216 CUP samples collected across multiple centers, the classifier identified the tissue of origin with 87% accuracy.

Fernandez et al. [18] (2012) reported an early study of DNA methylation fingerprints by examining 1628 human samples from several tissue types and a range of cancer entities. Using hierarchical clustering and supervised classification of Illumina 27K array data, the study found that DNA methylation profiles distinguished tissue types and tumor entities with consistent accuracy. These results support the view that DNA methylation patterns are specific to tissue type.

Zhang et al. [19] proposed computational rules to infer the tissue of origin of cancer using aberrant DNA methylation patterns. In this study, we trained decision tree classifiers and selected features with information-theory measures using TCGA methylation data. Their method identified tissue-specific methylation patterns that supported accurate classification while remaining interpretable in lung cancer and several other cancer types.

Xia et al. [20] studied minimalist methods for classifying the tissue of origin in cancer using DNA methylation data. The study assessed classifier performance across different counts of CpG features and found that LUAD and LUSC can be classified accurately using relatively small feature sets. The machine learning framework showed competitive performance with only 100 carefully chosen CpG probes.

Rauschert et al. [21] reviewed how machine learning has been applied in clinical epigenetics and discussed key issues in classifying cancers using DNA methylation data. The review noted that methylation-based classifier development should account for batch effects, choose and report feature selection methods clearly, address class imbalance, and include external validation.

The literature reviewed suggests that DNA methylation profiles, when analyzed with machine-learning and deep-learning methods, can classify lung cancer subtypes with good accuracy. The results indicate that random forest and deep neural network models can reach classification accuracy above 90% when distinguishing LUAD from LUSC. Performance is usually best when feature selection retains about 2000 to 10,000 CpG probes with strong class differences. Models trained on TCGA data should be tested on independent cohorts, since accuracy for the training source alone does not confirm that the model will hold up in other settings. Adding explanation tools such as LIME and SHAP can make the model outputs easier to interpret and may support clinical acceptance. These studies form the basis of our work. We train a deep neural network classifier on TCGA data and test it on external GEO datasets, and we include a SHAP-based analysis to interpret the model’s predictions.

## 3. Methodology

**Figure 1:**
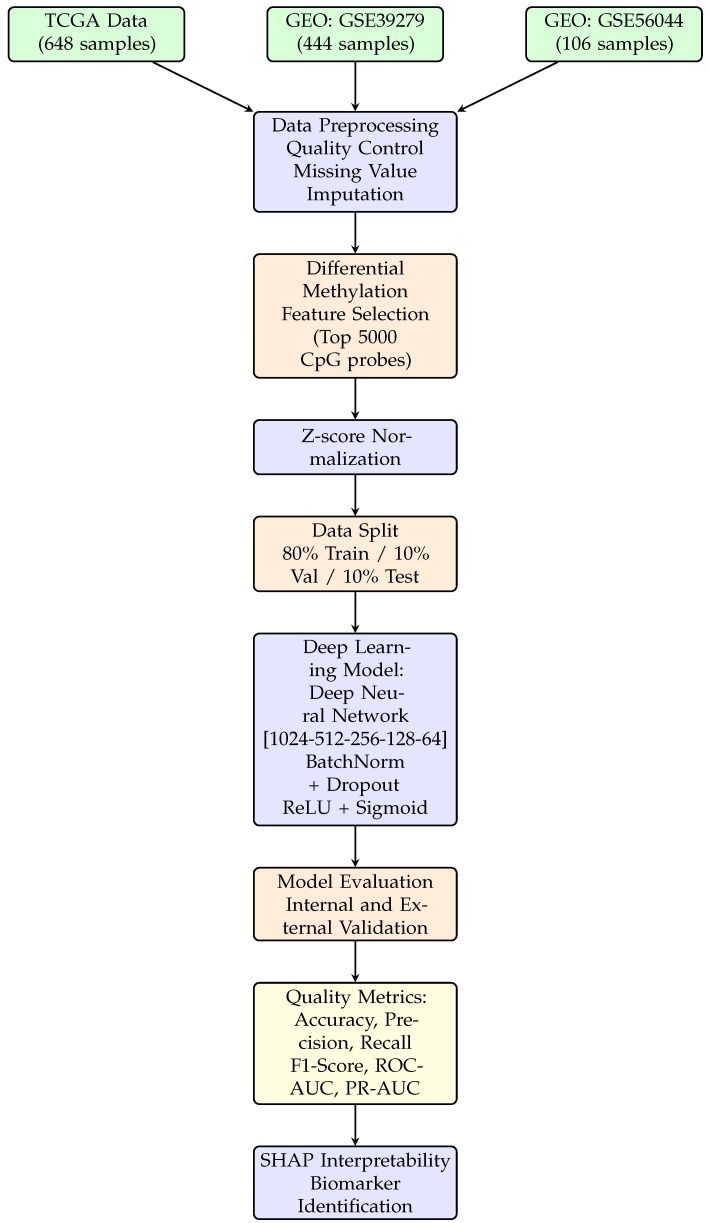
Methodology workflow for LUAD vs. LUSC classification using DNA methylation data. The pipeline includes data acquisition from TCGA and GEO repositories, preprocessing, feature selection, a deep learning model (five-layer DNN with ReLU activation and sigmoid output), multi-cohort validation, quality metrics evaluation (accuracy, precision, recall, F1-score, ROC-AUC, PR-AUC), and SHAP-based interpretability analysis.

This section presents the comprehensive methodology employed for developing a deep-learning-based classifier to distinguish between lung adenocarcinoma (LUAD) and lung squamous cell carcinoma (LUSC) using DNA methylation profiles. The methodology encompasses data acquisition from publicly available repositories, preprocessing and quality control procedures, differential methylation-based feature selection, deep neural network architecture design, model training with regularization techniques, external validation on independent cohorts, and interpretability analysis using SHAP. Figure 1 illustrates the complete workflow of the proposed approach.

### 3.1. Data Sources and Acquisition

The primary dataset for model development was obtained from The Cancer Genome Atlas (TCGA) program, a comprehensive cancer genomics resource maintained by the National Cancer Institute [22]. DNA methylation profiles were retrieved from the Genomic Data Commons (GDC) portal using the gdc-client tool for both LUAD (TCGA-LUAD project) and LUSC (TCGA-LUSC project) cohorts. The methylation data were generated using the Illumina Infinium HumanMethylation450 BeadChip platform (GPL13534), which interrogates the methylation status of over 450,000 CpG dinucleotide sites across the human genome [9].

Sample filtering was applied to retain only primary tumor samples, excluding adjacent normal tissue, recurrent tumors, and metastatic samples. The final TCGA cohort comprised 648 tumor samples, including 471 LUAD samples and 177 LUSC samples. Methylation values were represented as beta values (β), calculated as the ratio of methylated probe intensity to total intensity:

(1) $$\beta =\frac{M}{M+U+\alpha }$$

where *M* represents the methylated signal intensity, *U* represents the unmethylated signal intensity, and α is a constant offset (typically 100) to regularize β when both intensities are low.

For external validation, two independent datasets obtained from the Gene Expression Omnibus (GEO) repository:

GSE39279 (GEO1) is a dataset provided by the CURELUNG Consortium and comes from a multicenter European study on lung cancer. The dataset includes methylation profiles for 444 lung tumor samples, with 322 LUAD cases and 122 LUSC cases, measured using the Illumina HumanMethylation450 platform (GPL13534) [23].

GSE56044 (GEO2) is a smaller cohort with 106 lung tumor samples, including 83 LUAD and 23 LUSC cases, measured on the Illumina 450 K platform. It serves as an independent patient set used to check whether findings from the main analysis hold in a separate population [24].

Using several independent external validation cohorts allows a clear assessment of how well the model generalizes across different patient groups and across variation in laboratory processing conditions.

### 3.2. Data Preprocessing

Strict quality control procedures were applied to maintain the integrity of the data. CpG probes were excluded if more than 20% of samples had missing values, since this level of missingness may indicate unreliable measurements. In this filtering step, probes with technical issues were excluded, along with probes with single-nucleotide polymorphisms (SNPs) at the CpG site, and cross-reactive probes that could hybridize to more than one genomic location. Any remaining missing entries in the methylation data matrix were replaced with the median value using median imputation. For each CpG probe *j*, missing values were replaced with the median beta value calculated across all samples.

(2) $${\hat{\beta }}_{ij}=\mathrm{median}\left(\beta_{1j},\beta_{2j},\ldots ,\beta_{nj}\right)$$

where ${\hat{\beta }}_{ij}$ denotes the imputed value for sample *i* at probe *j*, and *n* denotes the number of samples with observed (non-missing) values at that probe. Median imputation was selected because it is less sensitive to outliers than mean imputation.

### 3.3. Feature Selection

Because methylation array datasets include about 450,000 CpG probes, feature selection was needed to focus on probes that best distinguish groups, reduce computation, and lower the risk of overfitting. Differential methylation analysis was used to select features. For each CpG probe, methylation differences between the LUAD and LUSC groups using Welch’s *t*-test were tested, which allows for unequal variances across groups. The test statistic was calculated as follows:

(3) $$t=\frac{{\bar{\beta }}_{\mathrm{LUAD}}-{\bar{\beta }}_{\mathrm{LUSC}}}{\sqrt{\frac{s_{\mathrm{LUAD}}^{2}}{n_{\mathrm{LUAD}}}+\frac{s_{\mathrm{LUSC}}^{2}}{n_{\mathrm{LUSC}}}}}$$

where ${\bar{\beta }}_{\mathrm{LUAD}}$ and ${\bar{\beta }}_{\mathrm{LUSC}}$ are the mean beta values for LUAD and LUSC groups, respectively, $s_{\mathrm{LUAD}}^{2}$ and $s_{\mathrm{LUSC}}^{2}$ are the sample variances, and $n_{\mathrm{LUAD}}$ and $n_{\mathrm{LUSC}}$ are the sample sizes.

The degrees of freedom for Welch’s *t*-test were approximated using the Welch–Satterthwaite equation:

(4) $$\nu \approx \frac{{\left(\frac{s_{\mathrm{LUAD}}^{2}}{n_{\mathrm{LUAD}}}+\frac{s_{\mathrm{LUSC}}^{2}}{n_{\mathrm{LUSC}}}\right)}^{2}}{\frac{{\left(s_{\mathrm{LUAD}}^{2}/n_{\mathrm{LUAD}}\right)}^{2}}{n_{\mathrm{LUAD}}-1}+\frac{{\left(s_{\mathrm{LUSC}}^{2}/n_{\mathrm{LUSC}}\right)}^{2}}{n_{\mathrm{LUSC}}-1}}$$

*p*-values were corrected for multiple testing using the Bonferroni correction:

(5) $$p_{\mathrm{adj}}=\min\left(p\times m,1\right)$$

where *m* is the total number of CpG probes tested.

The effect size for each probe was quantified using the absolute difference in mean methylation (delta beta):

(6) $$\Delta \beta =|{\bar{\beta }}_{\mathrm{LUAD}}-{\bar{\beta }}_{\mathrm{LUSC}}|$$

Probes were selected based on dual criteria: (1) statistical significance (adjusted *p*-value <0.05), and (2) biological relevance (Δβ>0.1). From the probes meeting these criteria, the top 5000 probes ranked by absolute delta beta were selected as features for the classification model.

Selected features were standardized using z-score normalization to ensure that all features contributed equally to the model and to facilitate gradient-based optimization:

(7) $$z_{ij}=\frac{\beta_{ij}-\mu_{j}}{\sigma_{j}}$$

where $z_{ij}$ is the normalized value for sample *i* at probe *j*, $\mu_{j}$ is the mean, and $\sigma_{j}$ is the standard deviation of probe *j* across training samples. Normalization parameters obtained from the training set are applied to both the validation and test sets to avoid data leakage.

Stratified sampling was used to divide the dataset into training (80%), validation (10%) and testing (10%) sets so that the proportions of classes would remain the same in each split. The training set was used in adjusting the parameters of the model during training. The validation set was used to determine when to terminate early training. The test set was not used until the end to ensure an objective measure of performance. The random seed was initialized to 42 to enable us to reproduce the data split.

### 3.4. Deep Neural Network Architecture

The classification model used a deep feedforward neural network with five hidden layers, with layer sizes decreasing from 1024 → 512 → 256 → 128 → 64. The funnel-shaped design gradually reduces the size of the input features, so the model can learn layered patterns, moving from raw methylation measurements to higher-level signals used for classification.

**Figure 2:**
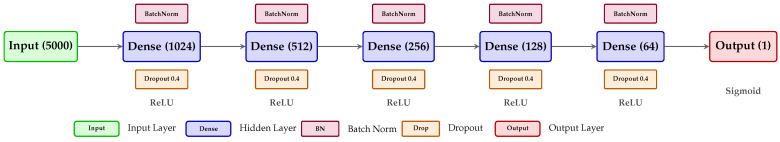
Architecture of the proposed deep feedforward neural network for LUAD/LUSC classification. The network consists of an input layer receiving 5000 CpG probe features, five hidden layers with progressively decreasing sizes (1024→512→256→128→64), and a single sigmoid output node. Each hidden layer incorporates batch normalization (purple) and dropout regularization with rate 0.4 (orange). ReLU activation is applied to all hidden layers, while sigmoid activation produces the final classification probability.

Figure 2 illustrates the complete architecture of the proposed deep neural network.

The model was based on a deep feedforward neural network with five hidden layers, whose sizes gradually decrease as 1024, 512, 256, 128, and 64. The model is capable of learning layered patterns, i.e., raw measurements of the methylation on the DNA molecules to the higher-order levels that will be used to classify the data.

The network structure for each hidden layer *l* can be expressed as

(8) $$h^{\left(l\right)}=f\left(\mathrm{BN}\left(W^{\left(l\right)}h^{\left(l-1\right)}+b^{\left(l\right)}\right)\right)$$

where $h^{\left(l\right)}$ is the output of layer *l*, $W^{\left(l\right)}$ and $b^{\left(l\right)}$ are the weight matrix and bias vector, BN denotes batch normalization, and *f* is the activation function.

The Rectified Linear Unit (ReLU) activation function was applied in all hidden layers:

(9) ReLU(x)=max(0,x)

ReLU was chosen for its computational efficiency and ability to mitigate the vanishing gradient problem that affects deep networks with saturating activation functions.

For the output layer, the sigmoid activation function was employed for binary classification:

(10) $$\sigma \left(x\right)=\frac{1}{1+e^{-x}}$$

producing output values $\hat{y}\in \left(0,1\right)$ interpretable as the probability of the LUSC class.

Batch normalization was applied after each linear transformation to stabilize and accelerate training [25]. For a mini-batch B of activations, batch normalization computes:

(11) $${\hat{x}}_{i}=\frac{x_{i}-\mu_{B}}{\sqrt{\sigma_{B}^{2}+\epsilon }}$$

(12) $$y_{i}=\gamma {\hat{x}}_{i}+\beta$$

where $\mu_{B}$ and $\sigma_{B}^{2}$ are the mini-batch mean and variance, ϵ is a small constant for numerical stability, and γ and β are learnable parameters.

Dropout regularization was incorporated after each hidden layer with a dropout rate of p=0.4 to prevent overfitting [26]. During training, each neuron output was randomly set to zero with probability *p*:

(13) $$h_{\mathrm{drop}}^{\left(l\right)}=h^{\left(l\right)}⊙m^{\left(l\right)},\;m_{i}^{\left(l\right)}∼\mathrm{Bernoulli}\left(1-p\right)$$

where ⊙ denotes element-wise multiplication and $m^{\left(l\right)}$ is a binary mask. During inference, dropout was disabled and outputs were scaled by (1−p).

Network weights were initialized using Xavier (Glorot) uniform initialization:

(14) $$W_{ij}∼U\left(-\sqrt{\frac{6}{n_{\mathrm{in}}+n_{\mathrm{out}}}},\sqrt{\frac{6}{n_{\mathrm{in}}+n_{\mathrm{out}}}}\right)$$

where $n_{\mathrm{in}}$ and $n_{\mathrm{out}}$ are the number of input and output units respectively. This initialization maintains activation variance across layers, facilitating stable training.

### 3.5. Computing Libraries and Environment

In a Linux system with NVIDIA GPU acceleration, all experiments were carried out using Python 3.10. The libraries used, along with their respective versions, were the following:

- **Deep learning:** PyTorch 2.0.1 with GPU acceleration via CUDA 11.8
- **Data processing:** NumPy 1.24.3, data manipulation and analysis with Pandas 2.0.2
- **Machine learning:** Scikit-learn 1.2.2 for data splitting, metric computation, and preprocessing
- **Statistical analysis:** SciPy 1.10.1 for differential methylation analysis using Welch’s *t*-test
- **Model interpretability:** SHAP 0.42.1 for the computation and visualization of Shapley values
- **Visualization:** Matplotlib 3.7.1, Seaborn 0.12.2 for plots and figure generation

The computational setup used an NVIDIA GPU to provide acceleration while training models. To enhance the reproducibility of results, all random seeds were set to 42.

### 3.6. Training Procedure

The network was trained to minimize the binary cross-entropy loss:

(15) $$L=-\frac{1}{N}\sum_{i=1}^{N}\left[y_{i}\log\left({\hat{y}}_{i}\right)+\left(1-y_{i}\right)\log\left(1-{\hat{y}}_{i}\right)\right]$$

where $y_{i}\in \left\{0,1\right\}$ is the true label (0 for LUAD, 1 for LUSC), ${\hat{y}}_{i}$ is the predicted probability, and *N* is the number of samples.

The Adam optimizer was employed for parameter updates, combining the benefits of momentum and adaptive learning rates [27]:

(16) $$m_{t}=\beta_{1}m_{t-1}+\left(1-\beta_{1}\right)g_{t}$$

(17) $$v_{t}=\beta_{2}v_{t-1}+\left(1-\beta_{2}\right)g_{t}^{2}$$

(18) $$\theta_{t+1}=\theta_{t}-\frac{\eta }{\sqrt{{\hat{v}}_{t}}+\epsilon }{\hat{m}}_{t}$$

where $g_{t}$ is the gradient, $m_{t}$ and $v_{t}$ are the first and second moment estimates, ${\hat{m}}_{t}$ and ${\hat{v}}_{t}$ are bias-corrected estimates, η=0.0001 is the learning rate, and $\beta_{1}=0.9$, $\beta_{2}=0.999$.

L2 regularization (weight decay) with the coefficient $\lambda =10^{-5}$ was applied to prevent overfitting:

(19) $$L_{\mathrm{total}}=L+\lambda \sum_{l}\left|\right|W^{\left(l\right)}{\left|\right|}_{2}^{2}$$

A ReduceLROnPlateau scheduler was employed to adaptively reduce the learning rate when validation loss plateaued. The learning rate was reduced by a factor of 0.5 if no improvement was observed for 10 consecutive epochs.

To prevent overfitting, early stopping was implemented with a patience of 20 epochs. Training was terminated if validation loss did not improve for 20 consecutive epochs, and the model checkpoint with the lowest validation loss was retained.

Training was conducted using the following hyperparameters:

- Batch size: 32
- Maximum epochs: 150
- Learning rate: $10^{-4}$
- Weight decay: $10^{-5}$
- Dropout rate: 0.4

All experiments were performed on an NVIDIA GPU using PyTorch as the deep learning framework.

### 3.7. Evaluation Metrics

Model performance was evaluated using standard binary classification metrics:

**Accuracy:**

(20) $$\mathrm{Accuracy}=\frac{TP+TN}{TP+TN+FP+FN}$$

**Precision (positive predictive value):**

(21) $$\mathrm{Precision}=\frac{TP}{TP+FP}$$

**Recall (sensitivity):**

(22) $$\mathrm{Recall}=\frac{TP}{TP+FN}$$

**F1-score:**

(23) $$F_{1}=2\cdot \frac{\mathrm{Precision}\cdot \mathrm{Recall}}{\mathrm{Precision}+\mathrm{Recall}}$$

**Area under ROC curve (AUC-ROC):** the ROC curve shows how the true positive rate and false positive rate change as the classification threshold is adjusted. AUC-ROC was used as a threshold-independent measure of how well the model separates the two classes, where TP, TN, FP, and FN denote true positives, true negatives, false positives, and false negatives, respectively, with LUSC as the positive class.

### 3.8. External Validation Strategy

To rigorously assess model generalizability, a comprehensive cross-dataset validation framework was employed. Six experimental configurations were evaluated:

1. **TCGA → TCGA:** internal validation using held-out TCGA test set
2. **TCGA → GEO1:** model trained on TCGA, validated on GSE39279
3. **TCGA → GEO2:** model trained on TCGA, validated on GSE56044
4. **GEO1 → TCGA:** model trained on GSE39279, validated on TCGA
5. **GEO1 → GEO1:** internal validation on GSE39279
6. **GEO1 → GEO2:** model trained on GSE39279, validated on GSE56044

For external validation, CpG probes and normalization settings selected during training were applied to the external dataset. Only probes found in both the training and validation datasets were included.

### 3.9. Model Interpretability with SHAP

To improve transparency in the model and support the search for potential methylation biomarkers, SHAP (Shapley Additive Explanations) analysis [28] is applied. SHAP values measure how much each feature shifts a model’s prediction for a specific case, using a cooperative game theory method to split the prediction across features.

For a model *f* and input x, the SHAP value $\phi_{j}$ for feature *j* is defined as

(24) $$\phi_{j}=\sum_{S\subseteq N\setminus \{j\}}\frac{|S|!(|N|-|S|-1)!}{\left|N\right|}\left[f\left(S\cup \left\{j\right\}\right)-f\left(S\right)\right]$$

where N refers to the full set of features; S is any subset that does not include feature j; and f(S) is the model’s prediction when it is run using only the features in S.

SHAP library’s DeepExplainer method was used to compute SHAP values for the deep neural network in a time-efficient way. For DeepExplainer’s reference distribution, a background dataset of 100 training samples selected at random was used. The SHAP analysis produced several outputs that fit together and supported the interpretation of the results. We estimated global feature importance by taking the average absolute SHAP value across all samples, which allowed us to identify the CpG probes with the strongest overall impact on the classification results. We created summary plots to show how each feature value is linked to its effect on the model’s predictions. Dependence (scatter) plots were made to check for interactions between the top features and their methylation values. The CpG probes ranked highest by SHAP were then reviewed for biological meaning. Then, these probes were mapped to gene databases to identify the related genes and check what is known about those genes in lung cancer.

## 4. Results

This section summarizes the experimental results for the deep learning model used to classify LUAD versus LUSC. The evaluation includes internal validation using held-out test sets, external validation across datasets by comparing TCGA and GEO cohorts, and SHAP interpretability analysis to determine which CpG biomarkers have the strongest influence on the model.

### 4.1. Dataset Characteristics

After quality filtering, the TCGA dataset included 648 primary tumor samples: 471 lung adenocarcinoma (LUAD, 72.7%) and 177 lung squamous cell carcinoma (LUSC, 27.3%). Following the 80/10/10 stratified split, the training set contained 518 samples, with 65 samples each allocated to validation and test sets. The class distribution in the test set was 37 LUAD and 28 LUSC samples.

The GSE39279 dataset from the CURELUNG Consortium included 444 tumor samples: 322 LUAD (72.5%) and 122 LUSC (27.5%). When used for training in the swapped experiment, the 80/10/10 split yielded 355 training, 44 validation, and 45 test samples.

The GSE56044 dataset included 106 tumor samples, of which 83 were LUAD (78.3%) and 23 were LUSC (21.7%). This dataset was used exclusively for external validation.

### 4.2. Experiment Set 1: TCGA-Trained Model

The deep neural network was trained on TCGA data and showed strong performance on the held-out TCGA test set (n = 65). Table 1 summarizes the classification metrics for the internal TCGA cohort.

The confusion matrix shows 35 true negatives, where LUAD was correctly classified, and 28 true positives, where LUSC was correctly classified. There were 2 false positives, meaning that LUAD was classified as LUSC, and 0 false negatives, meaning that no LUSC samples were classified as LUAD. A recall of 100% means that all LUSC cases in the dataset were classified correctly, which is important in clinical practice because treatment decisions depend on accurate subtype identification.

**Figure 3:**
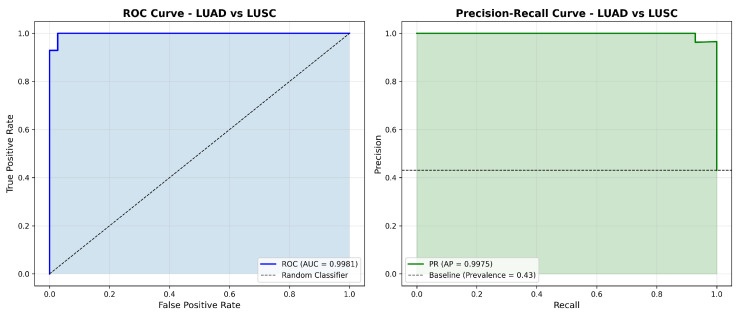
ROC curve (**left**) and precision–recall curve (**right**) for the TCGA-trained model on internal test set. The ROC-AUC of 0.9981 and PR-AUC of 0.9975 shows the excellent classification performance.

Figure 3 presents the ROC and precision–recall curves for the TCGA-trained model, demonstrating the strong discriminative performance with AUC values approaching 1.0.

**Figure 4:**
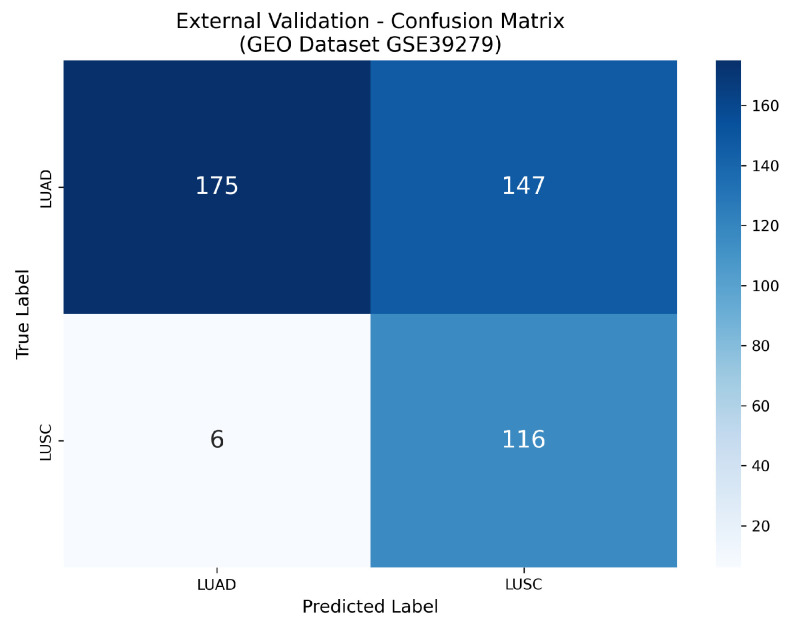
Confusion matrix for TCGA-trained model on TCGA test set. TN = 35, FP = 2, FN = 0, TP = 28.

Confusion matrix visualization for the TCGA internal validation can be seen in Figure 4.

The TCGA-trained model was evaluated using the independent GSE39279 dataset, which includes 444 samples. The model demonstrated lower performance on this external dataset compared to its results within the TCGA cohort. Results shows the model generalizability on external dataset. A summary of the evaluation results is presented in Table 2.

TN = 175, FP = 147, FN = 6, TP = 116 can be seen in confusion matrix. The model demonstrated a high sensitivity of 95.08% in identifying LUSC cases. However, the specificity was considerably lower at 54.35%, which led to a significant number of LUAD samples being misclassified as LUSC. Despite the reduction in specificity, the model got a relatively high ROC-AUC value of 0.9391. This indicates that the probabilistic outputs of the model continued to provide strong discriminative capability, even though the performance based on the classification threshold was affected.

External validation on the GSE56044 dataset (n = 106) yielded intermediate performance. The results are presented in Table 3.

The confusion matrix revealed TN = 64, FP = 19, FN = 0, TP = 23. Similar to GEO1 validation, the model achieved perfect recall (100%) for LUSC detection while showing reduced specificity. The exceptionally high ROC-AUC of 0.9932 indicates that probability calibration issues, rather than fundamental discriminative capability, account for the accuracy reduction.

### 4.3. Experiment Set 2: GEO1-Trained Model

To investigate the impact of training data source on generalization, a second model was trained on the GSE39279 (GEO1) dataset and validated on TCGA and GSE56044 cohorts.

The GEO1-trained model achieved strong performance on its internal test set (n = 45). Table 4 presents the results.

The confusion matrix showed TN = 33, FP = 0, FN = 3, and TP = 9, with the test set containing 33 LUAD and 12 LUSC samples. The model achieved perfect specificity (100%) with no false positives, though recall was lower at 75% due to 3 LUSC samples being misclassified.

The GEO1-trained model was validated on the complete TCGA cohort of 722 samples (excluding samples used in the original TCGA training). Table 5 summarizes the external validation results.

The confusion matrix revealed TN = 389, FP = 8, FN = 72, TP = 253, with the validation set containing 397 LUAD and 325 LUSC samples. The model demonstrated excellent specificity (97.98%) and precision (96.93%), indicating reliable LUAD classification, with moderate sensitivity (77.85%) for LUSC detection.

**Figure 5:**
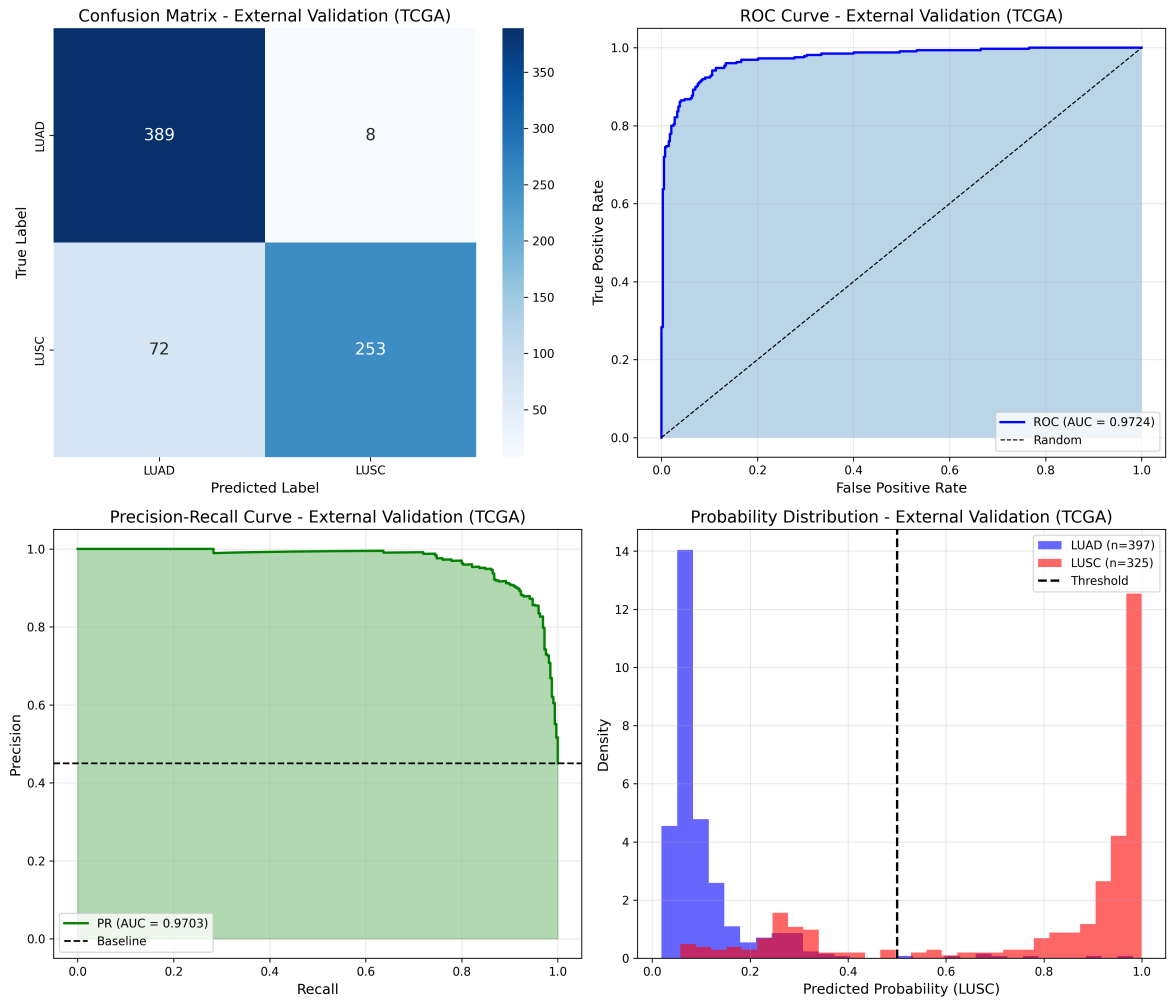
External validation results of GEO1-trained model on TCGA dataset (n = 722), showing ROC curve, precision–recall curve, confusion matrix, and probability distributions.

Figure 5 presents the comprehensive validation results for the GEO1-trained model on TCGA data.

The GEO1-trained model was evaluated using the GSE56044 dataset (n = 106). Initial evaluation revealed probability calibration shift, prompting threshold optimization. Table 6 presents the results with the optimal threshold of 0.29.

The confusion matrix with optimal threshold showed TN = 82, FP = 1, FN = 4, and TP = 19. This represented the best external validation performance across all experiments, demonstrating that the GEO1-trained model generalized well to independent GEO data.

**Figure 6:**
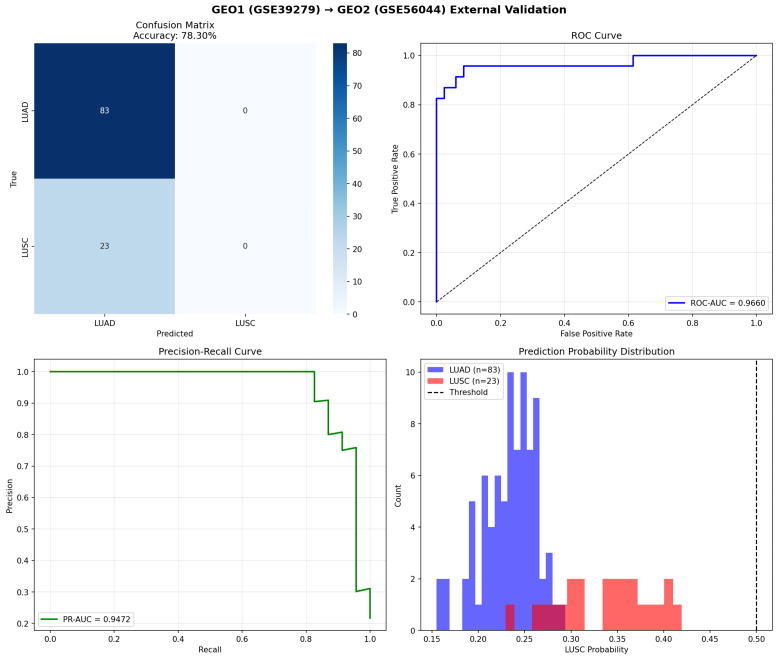
The The GEO1-trained model was externally validated using the GSE56044 dataset, which has 106 samples. To account for probability calibration shift, the classification threshold was optimized and set to 0.29. The dotted diagonal line in the ROC curve represents random classifier performance (AUC = 0.5).

Figure 6 shows the validation results including the probability calibration analysis.

A comprehensive summary of all six experimental configurations, enabling direct comparison of internal and external validation performance, can be seen in Table 7.

Key observations from the comprehensive comparison include the following:

1. **Internal Validation:** the internal validation of the both models was very good. The model which was trained on TCGA, achieved an accuracy of 96.92%, and the model which was trained on GEO1 was 93.33%.
2. **Cross-Dataset Generalization:** the GEO1-trained model demonstrated superior cross-dataset generalization (88.92% on TCGA, 95.28% on GEO2) compared to the TCGA-trained model (65.54% on GEO1, 82.08% on GEO2).
3. **ROC-AUC Preservation:** despite the differences in the accuracy of the experiments, the models obtained the same results with the ROC-AUC value of more than 0.93. This finding indicates that the probabilistic forecasts generated by the models were characterized by a high rate of discriminative ability in the different datasets that were considered.
4. **Sensitivity–Specificity Trade-off:** the TCGA-trained model exhibited high sensitivity but lower specificity on external data, while the GEO1-trained model showed the opposite pattern with high specificity and moderate sensitivity.

### 4.4. Training Dynamics

**Figure 7:**
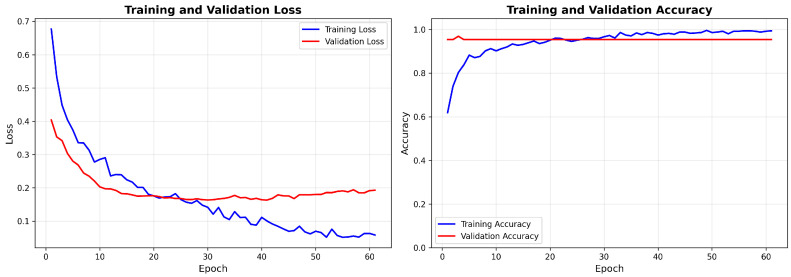
Training and validation curves for the TCGA-trained model, showing loss (**left**) and accuracy (**right**) progression across epochs. Early stopping was triggered when validation loss ceased improving.

Figure 7 presents the learning curves during model training, showing the convergence of training and validation loss and accuracy metrics over epochs.

**Figure 8:**
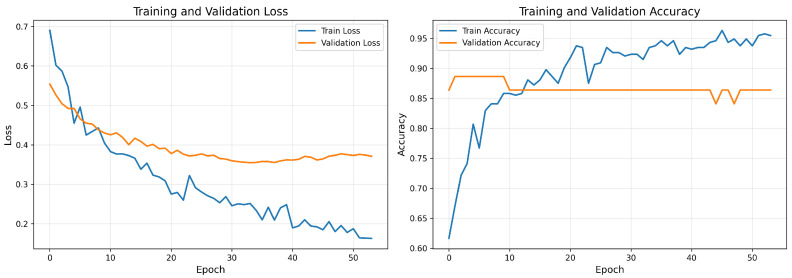
Training and validation curves for the model trained on GEO (GSE39279) showing the loss (**left**) and accuracy (**right**) across epochs. The validation metrics have a touch higher variance because the dataset is smaller than TCGA.

Figure 8 presents the corresponding learning curves for the GEO-trained model (GSE39279 dataset).

The learning curves show no signs of overfitting and demonstrate pretty stable convergence, with validation metrics closely following training metrics throughout the optimization process. Between the two training scenarios, the TCGA model shows smoother convergence due to the larger sample size, while the GEO model achieves comparable final performance despite using a smaller training dataset.

### 4.5. Cross-Validation Analysis

We performed stratified five-fold cross-validation on the TCGA dataset to measure model robustness and avoid possible overfitting due to reliance on a single train/test split. Results from the cross-validation can be found in Table 8.

We obtained 95.53% ± 1.49% for mean accuracy, 97.11% ± 2.33% for mean precision, 92.58% ± 2.61% for mean recall, 94.75% ± 1.76% for mean F1-score, and 0.9832 ± 0.0101 for mean ROC-AUC. The low standard deviations for the aforementioned metrics indicate that the performance of the model is high and not dependent on the choice of data split. Thus, the model showcases a high degree of generalization.

**Figure 9:**
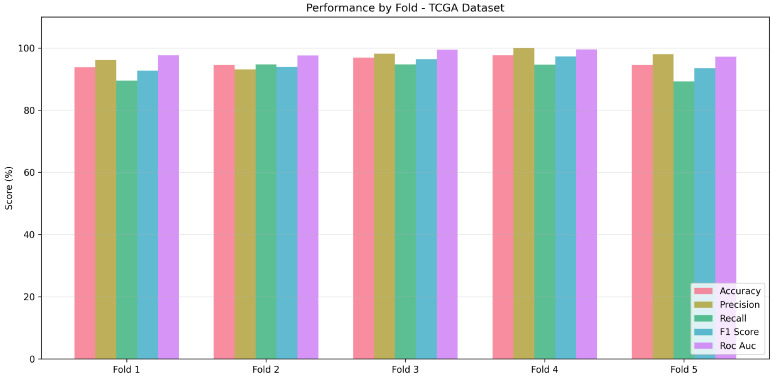
Five-fold cross-validation performance on TCGA dataset. Each grouped bar represents one fold, with metrics including accuracy, precision, recall, F1-score, and ROC-AUC. The consistent performance across folds demonstrates model stability.

Figure 9 visualizes the performance metrics across all five folds, illustrating the consistency of the classification performance.

### 4.6. Ablation Study

An ablation study assessed individual architectural components contributing to classification performance. Four key components were systematically tested: network depth, dropout rate, batch normalization, and number of input features. The results are presented in Table 9.

**Figure 10:**
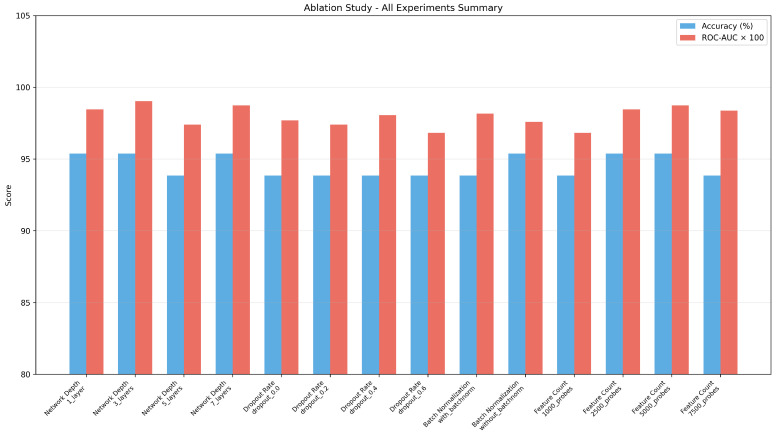
Ablation study summary comparing accuracy and ROC-AUC across different architectural configurations. The baseline configuration (5 layers, dropout 0.4, batch normalization, 5000 probes) demonstrates competitive performance across all metrics.

The ablation study reveals several insights: (1) network depth shows negligible impact on accuracy, with all configurations exceeding 93% accuracy; (2) a dropout rate of 0.4 provides optimal regularization, achieving the highest ROC-AUC among dropout variants; (3) batch normalization yields improved discriminative capability (ROC-AUC 0.9817 vs. 0.9759); and (4) using 5000 CpG probes achieves the best ROC-AUC (0.9875), with results plateauing beyond this threshold. Figure 10 presents a comprehensive visualization of all ablation experiments.

### 4.7. SHAP Interpretability Analysis

SHAP (Shapley Additive Explanations) analysis was conducted to identify the most influential CpG probes contributing to classification decisions. The analysis was performed on both the TCGA-trained and GEO1-trained models.

**Figure 11:**
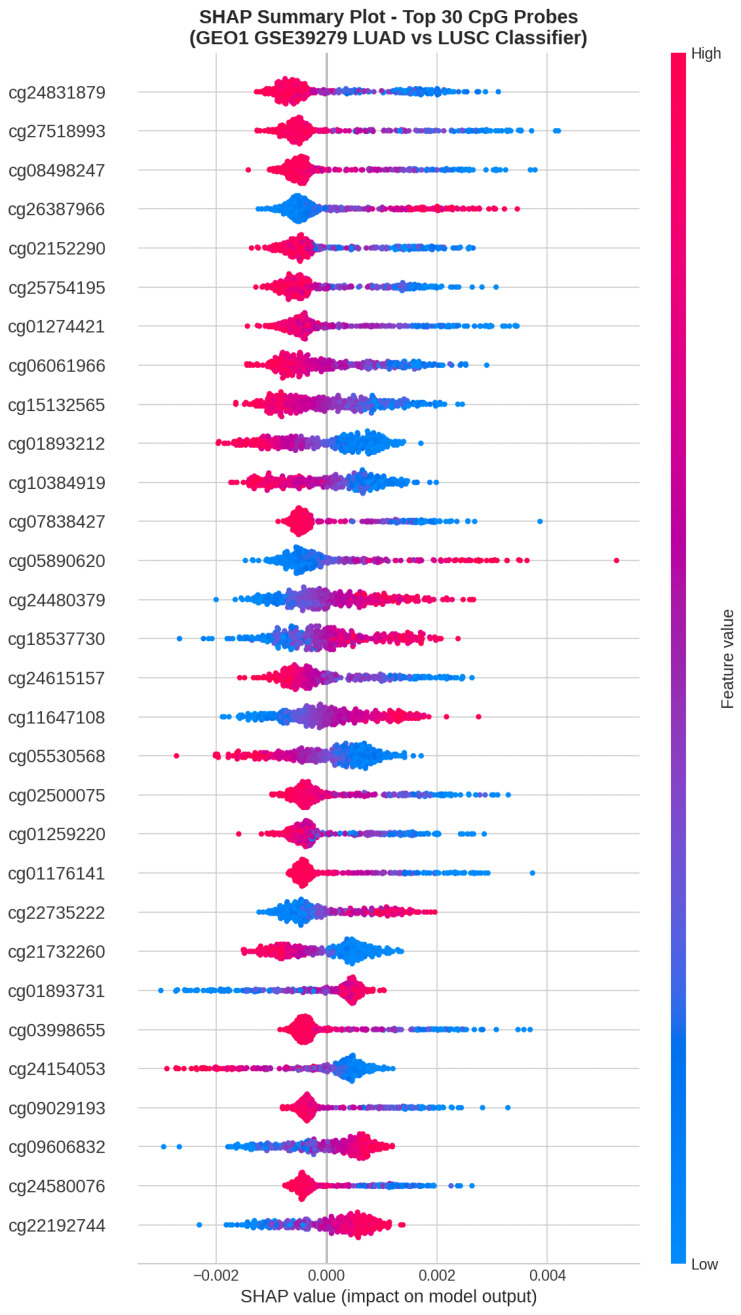
SHAP summary beeswarm plot showing the top 20 most influential CpG probes for the TCGA-trained model. Each point represents a sample, with color indicating feature value (red = high methylation, blue = low) and horizontal position indicating SHAP value (positive = LUSC, negative = LUAD).

Figure 11 presents the SHAP summary beeswarm plot for the top 20 most influential CpG probes in the TCGA-trained model.

The beeswarm plot reveals distinct methylation patterns associated with each cancer subtype. For many top probes, high methylation values (red) consistently push predictions toward LUSC (positive SHAP values), while low methylation values (blue) favor LUAD classification.

**Figure 12:**
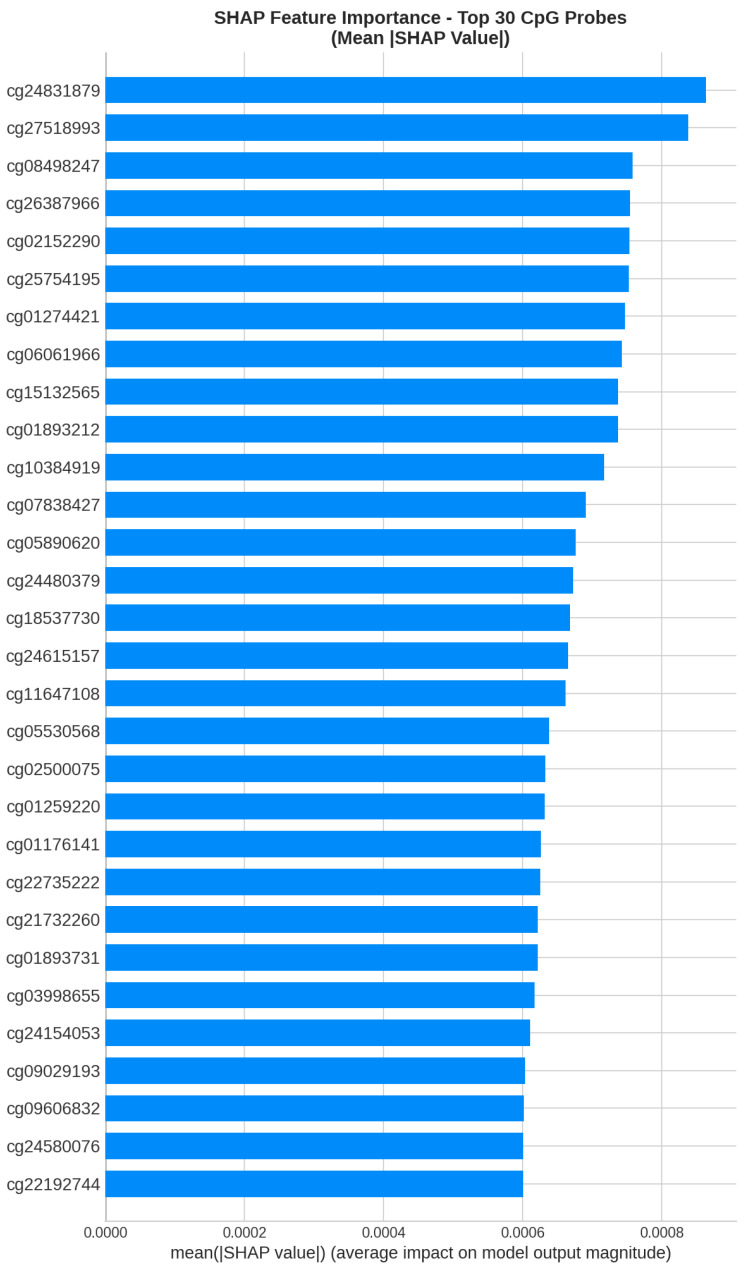
Global feature importance ranking for the TCGA-trained model based on mean absolute SHAP values. The top 20 CpG probes contributing most to classification decisions are displayed.

Figure 12 shows the global feature importance ranking based on mean absolute SHAP values.

**Figure 13:**
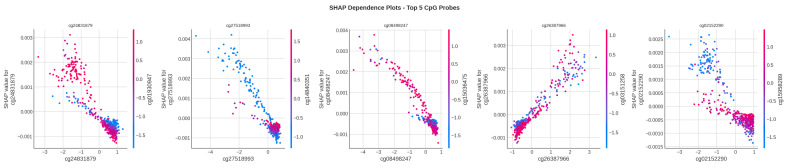
SHAP dependence plots for the top 5 most influential CpG probes. Each plot shows the relationship between the probe’s methylation value (x-axis) and its SHAP contribution (y-axis), with color indicating interaction effects.

Figure 13 presents SHAP dependence plots for the top 5 most important CpG probes, revealing the relationship between methylation levels and their impact on predictions.

The dependence plots show that the relationship between the values of the methylation and SHAP is characterized by monotony in most of the top probes and that the difference in the levels of the methylation between the LUAD and LUSC in these loci are consistent and meaningful.

**Figure 14:**
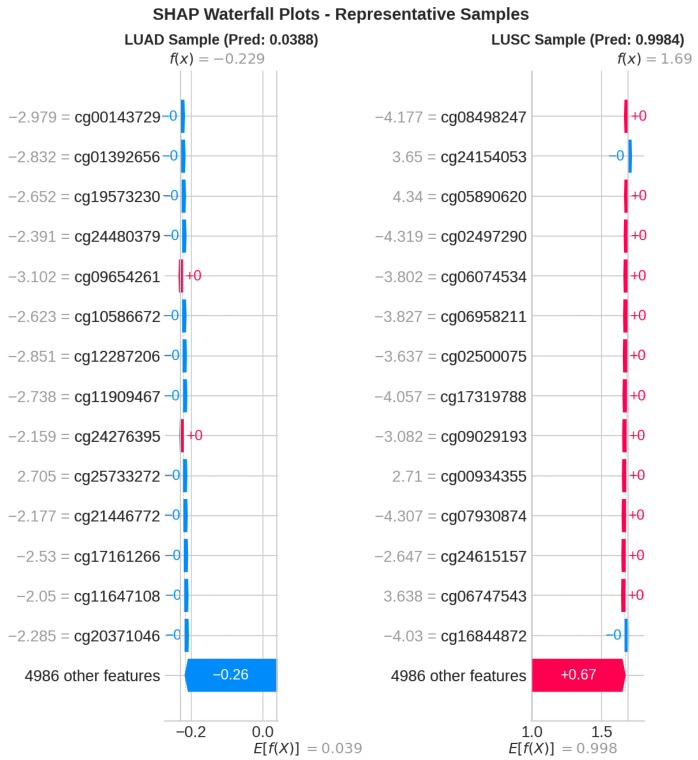
The SHAP waterfall plots portray individual sample predictions in terms of feature contributions. Each feature’s contribution to the final prediction score is depicted in these plots by showing how each feature alters the base value. In these plots, f(x) refers to the predicted output of the model (log-odds or probability) for an individual sample *x*, while E[f(x)] denotes the expected value (base value), which is the average model prediction across the training set. Features are arranged from top to bottom with the most to least absolute contribution, where red bars indicate positive contributions (contributing to LUSC) and blue bars indicate negative contributions (contributing to LUAD).

Figure 14 displays SHAP waterfall plots for individual sample predictions. These plots illustrate the contribution of specific CpG probe values to the overall classification outcomes.

**Figure 15:**
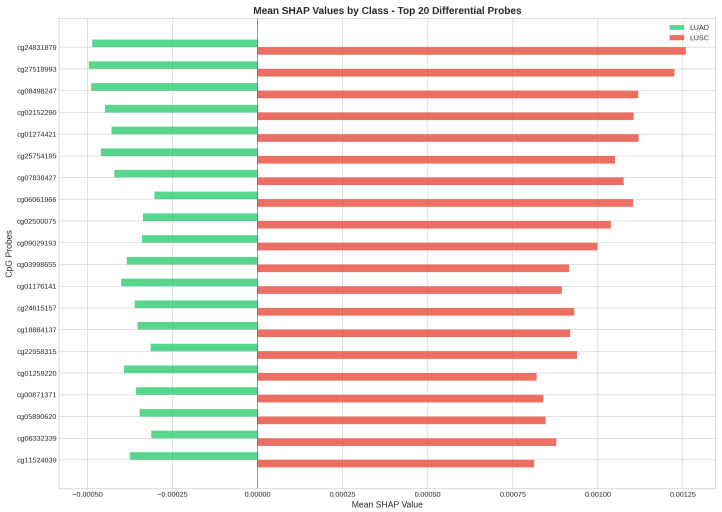
Comparison of mean SHAP values between LUAD and LUSC samples for the top 20 CpG probes. This visualization identifies probes that consistently distinguish between the two cancer subtypes.

Figure 15 compares mean SHAP values between LUAD and LUSC samples, highlighting the discriminative CpG probes for each class.

Comparing the two classes shows a clear symmetry: probes that increase support for LUSC tend to decrease support for LUAD, which supports the view that the selected biomarkers distinguish between the two diagnoses.

**Figure 16:**
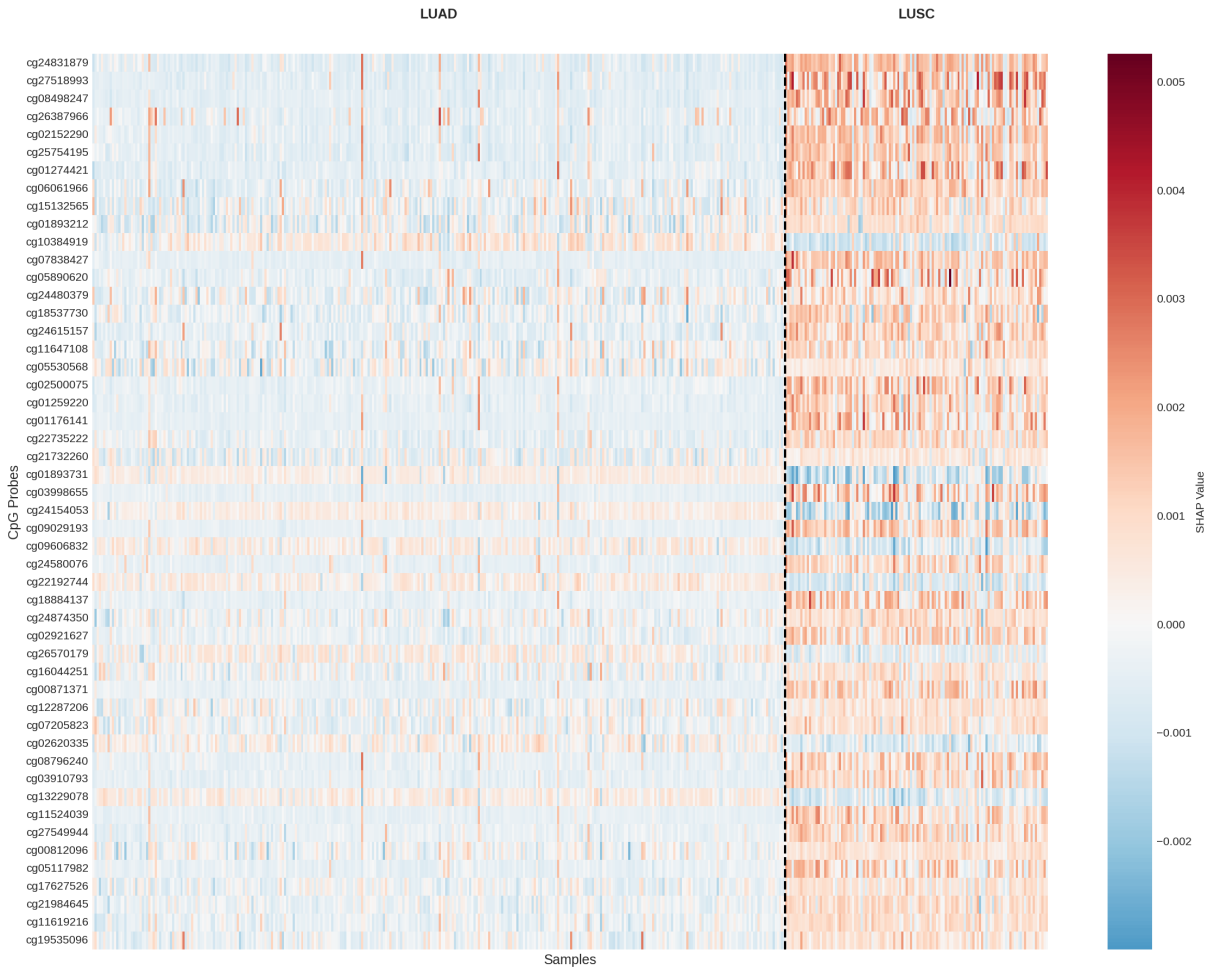
SHAP value heatmap for the top 50 CpG probes across samples, clustered by SHAP patterns. The heatmap reveals consistent patterns distinguishing LUAD from LUSC samples.

Figure 16 presents a heatmap of SHAP values for the top 50 CpG probes across samples, providing a comprehensive view of feature contributions.

The SHAP heatmap demonstrates clear clustering of samples by cancer subtype based on their SHAP value patterns, validating the model’s ability to learn biologically relevant methylation signatures.

## 5. Discussion

In this study, a deep neural network classifier was trained and tested to distinguish lung adenocarcinoma (LUAD) from lung squamous cell carcinoma (LUSC) using genome-wide DNA methylation data. Evaluation across multiple patient cohorts clarifies the capabilities and limitations of methylation-based cancer subtyping using deep learning methods.

The deep neural network trained on TCGA data achieved strong internal validation performance with 96.92% accuracy and ROC-AUC of 0.9981. This demonstrates that DNA methylation profiles contain sufficient discriminative information to distinguish LUAD from LUSC. Table 10 presents a comparison of our methodology with recent state-of-the-art approaches.

Our approach achieves competitive internal validation accuracy compared to these benchmarks. While some studies report higher accuracies, they often lack rigorous external validation on independent cohorts. The distinctive contribution of our work lies in the systematic cross-dataset validation using three independent cohorts (TCGA, GSE39279, GSE56044), all profiled using the Illumina HumanMethylation450 BeadChip (GPL13534), ensuring probe compatibility and comparable methylation measurements.

The experiments conducted on cross-dataset validation stated the process of generalization of the model. The model trained on TCGA records exhibited consistent ROC-AUC (receiver-operating characteristic–area under curve) values on foreign GEO (Gene Expression Omnibus) records (0.9391 on GSE39279, 0.9932 on GSE56044) but the values of accuracy were 65.54% and 82.08%, respectively. The explanation for the difference on ROC-AUC and the value of accuracy is that the greatest problem in cross-dataset generalization is most probably in the calibration of the probabilities rather than the discriminatory power for the values presented.

The GEO model provided external validation accuracy of TCGA (88.92%) data in the reverse direction than GEO (65.54%), it is likely that the major contributing factors are the sample size and population. These findings are similar to Rauschert et al. [21], where the most prominent challenges in the clinical use of methylation-based classifiers were batch effects and the specific cohorts.

The gap of performance in the observed case is attributed to the batch effects from the differing sample processing, array construction lots, and varying laboratory workflows. We minimized these using differential methylation analysis, feature selection and z-score normalization. Advanced cross-dataset calibration could be achieved using ComBat [29] style harmonization in future initiatives.

Correct histological subtyping of NSCLC has important consequences for patient care. LUAD and LUSC respond to different treatments and have different molecular characteristics [5]. Adenocarcinomas have EGFR mutations and ALK rearrangements, which are responsive to the currently approved tyrosine kinase inhibitors [6]. If adenocarcinomas are misclassified as something else, patients lose out on targeted therapies; on the other hand, patients may be exposed to unnecessary treatments.

The 100% recall score for the internal validation and 95% to 100% recall for the external validation on LUSC detection speaks to the high clinical relevance of the model as it shows that the model will not miss any of the squamous cell carcinoma patients. On the other hand, the LUAD over-classification as LUSC, which affects external data sets, and therefore LUSC under-classification, requires attention before the model can be used in clinical practice. The clinically useful predictive outputs can also be shown with the ROC-AUC scores, which are all over 0.93, and can be paired with confidence intervals to support pathologists in cases where there is uncertainty.

Our methylation-based classifier has potential advantages over current histopathological diagnosis using immunohistochemistry (IHC) panels, including possible objectivity, quantitative confidence scoring, and the ability to work with small biopsies. However, the additional infrastructure needed, the longer turnaround time (3–5 days versus 1–2 days for IHC) and the higher price are relevant counterpoints. The best use case may be as an adjunct for challenging cases where the morphology and IHC are inconclusive. As Lai et al. [30] noted, this is where the clinical relevance is, and it will need significant validation and standardization.

To facilitate an explanation of the reasoning, we used SHAP for the explanation of the reasoning. The analysis shows the model is able to capture relevant biology and not some irrelevant features of the dataset, as the cross-cohort separation of samples is consistently observed. The equal and opposite distributions of SHAP values for the top features selected for LUAD and LUSC samples suggests that the chosen markers of methylation are in fact capturing some of the relevant biology that distinguishes the two cancer classifications.

The CpG probes most impactful, as determined by SHAP analysis, represent potential candidates for further biological exploration. Changes in DNA methylation within certain regions of the genome have caused tumor suppressor genes to become silenced, while oncogenic pathways are activated in lung cancer [7,8]. The overlap of SHAP features and proven differential methylation analysis candidates suggests the model exhibits a choice-making feature that embodies genuine biological relevance. These probes capture the unique developmental pathways of LUAD and LUSC, with the former being alveolar adenocarcinoma, and the latter, squamous carcinoma of bronchial basal cells after squamous metaplasia.

The use of differential methylation analysis to select 5000 CpG probes enabled the acquisition of significant biological insights while achieving a form of dimensionality reduction. This mirrors the prior work of Xia et al. [20], where it was shown that accurate classifications may be derived from a reduced feature set. The differential methylation approach, as opposed to supervised techniques (e.g., LASSO, recursive feature elimination), was deliberate in not seeking an optimal solution due to the risk of overfitting. Welch’s *t*-test, while considering unequal variances, identifies probes that carry a significant statistical difference. Biologically relevant probes are yielded as a result of the dual criteria of statistical significance (Bonferroni-corrected *p*-value < 0.05) and effect size (Δβ>0.1).

A five hidden-layer deep neural network (1024, 512, 256, 128, 64) was created to learn intricate patterns of methylation. Loss during training was countered using additional techniques such as early stopping and the best model checkpointing, batch normalization and dropout (0.4). The architecture and the hyperparameters were chosen based on preliminary empirical results. Therefore, Bayesian hyperparameter optimization could characterize some improvements in the future.

The validation of external datasets showed a considerable shift in probability calibration that required a reduction in the threshold on the positive side from 0.5 to 0.29. This is a typical scenario for predictive analytics on separate (disjoint) datasets. A calibration technique such as isotonic regression or Platt scaling could be instrumental to improve interdependent dataset model reliability in future studies.

The following limitations should be noted: (1) the model’s utility in the clinic will have to be validated prospectively as this study relies on retrospective cohort data from the TCGA and GEO repositories, (2) model training was done exclusively on data from the Illumina HumanMethylation450 platform and generalizability to other platforms is not known, (3) the model may be affected by the imbalanced classes as LUAD is 70–78% of the cases, (4) when the model is applied to a dataset, the inter-dataset shifts in probability calibration will likely require thresholding to be adjusted, and (5) the CpG biomarkers lack the experimental validation of bisulfite sequencing and lack correlation with expression data. These limitations will form the basis of future studies, including prospective clinical trials and the experimental validation of the metrics.

## 6. Conclusions

This study shows that deep neural networks (DNNs) trained on genome-wide DNA methylation profiles can distinguish between lung adenocarcinoma (LUAD) and lung squamous cell carcinoma (LUSC). The notable findings are the following:

- The model trained on TCGA data obtained an accuracy of 96.92% and achieved 0.9981 ROC-AUC during internal validation.
- The model trained on GEO data exhibited solid and robust cross dataset generalization with an accuracy of 88.92% and a ROC-AUC of 0.9724 when validated on TCGA data.
- Every experimental setup persisted with ROC-AUC values above 0.93, which shows they had the ability to consistently distinguish across different cohorts.
- LUSC detection across the different datasets exhibited high sensitivity (95–100%) and shows that these models may have clinical utility.
- CpG biomarkers that drive classification decisions were identified and explained through SHAP analysis.

Prospective clinical validation, cross-dataset calibration, and integration of identified methylation biomarkers to the models for clinical utility will be the focus of future work.

## Figures and Tables

**Table 1:** Classification performance of TCGA-trained model on TCGA test set (n = 65).

Metric	Value
Accuracy	96.92%
Precision	93.33%
Recall (sensitivity)	100.00%
F1-score	96.55%
Specificity	94.59%
ROC-AUC	0.9981
PR-AUC	0.9975

**Table 2:** External validation of TCGA-trained model on GSE39279 (n = 444).

Metric	Value
Accuracy	65.54%
Precision	44.11%
Recall (sensitivity)	95.08%
F1-score	60.26%
Specificity	54.35%
ROC-AUC	0.9391
PR-AUC	0.8972

**Table 3:** External validation of TCGA-trained model on GSE56044 (n = 106).

Metric	Value
Accuracy	82.08%
Precision	54.76%
Recall (sensitivity)	100.00%
F1-score	70.77%
Specificity	77.11%
ROC-AUC	0.9932
PR-AUC	0.9832

**Table 4:** Classification performance of GEO1-trained model on GEO1 test set (n = 45).

Metric	Value
Accuracy	93.33%
Precision	100.00%
Recall (sensitivity)	75.00%
F1-score	85.71%
Specificity	100.00%
ROC-AUC	0.9773
PR-AUC	0.9501

**Table 5:** External validation of GEO1-trained model on TCGA dataset (n = 722).

Metric	Value
Accuracy	88.92%
Precision	96.93%
Recall (sensitivity)	77.85%
F1-score	86.35%
Specificity	97.98%
ROC-AUC	0.9724
PR-AUC	0.9703

**Table 6:** External validation of GEO1-trained model on GSE56044 with optimal threshold (n = 106).

Metric	Value
Accuracy	95.28%
Precision	95.00%
Recall (sensitivity)	82.61%
F1-score	88.37%
Specificity	98.80%
ROC-AUC	0.9660
PR-AUC	0.9472

**Table 7:** All experiments classification performance summary.

Experiment	n	Accuracy	Precision	Recall	F1-Score	ROC-AUC
Experiment Set 1: TCGA-Trained Model						
1. TCGA → TCGA	65	96.92%	93.33%	100.00%	96.55%	0.9981
2. TCGA → GEO1	444	65.54%	44.11%	95.08%	60.26%	0.9391
3. TCGA → GEO2	106	82.08%	54.76%	100.00%	70.77%	0.9932
Experiment Set 2: GEO1-Trained Model						
4. GEO1 → TCGA	722	88.92%	96.93%	77.85%	86.35%	0.9724
5. GEO1 → GEO1	45	93.33%	100.00%	75.00%	85.71%	0.9773
6. GEO1 → GEO2 *	106	95.28%	95.00%	82.61%	88.37%	0.9660

* With optimal threshold = 0.29.

**Table 8:** Five-fold cross-validation results on TCGA dataset (n = 648).

Metric	Fold 1	Fold 2	Fold 3	Fold 4	Fold 5
Accuracy (%)	93.85	94.62	96.92	97.67	94.57
Precision (%)	96.23	93.10	98.18	100.00	98.04
Recall (%)	89.47	94.74	94.74	94.64	89.29
F1-score (%)	92.73	93.91	96.43	97.25	93.46
ROC-AUC	0.9772	0.9760	0.9952	0.9956	0.9721

**Table 9:** Ablation study results on TCGA dataset.

Factor	Configuration	Accuracy (%)	ROC-AUC
Network Depth	1 layer (512)	95.38	0.9846
	3 layers (512→256→128)	95.38	0.9903
	5 layers (baseline)	93.85	0.9739
	7 layers	95.38	0.9875
Dropout Rate	0.0 (none)	93.85	0.9768
	0.2	93.85	0.9739
	0.4 (baseline)	93.85	0.9807
	0.6	93.85	0.9681
Batch Norm	With (baseline)	93.85	0.9817
	Without	95.38	0.9759
Feature Count	1000 probes	93.85	0.9681
	2500 probes	95.38	0.9846
	5000 probes (baseline)	95.38	0.9875
	7500 probes	93.85	0.9836

**Table 10:** Comparison with state-of-the-art DNA methylation classification methods for lung cancer.

Study	Method	Task	Accuracy	External Validation
Jurmeister et al. (2019) [13]	ANN	LUSC vs. HNSCC	96.4%	Single cohort
Modhukur et al. (2021) [11]	Random forest	Pan-cancer (24 types)	99.0%	Not reported
Zheng & Xu (2020) [12]	DNN	Pan-cancer origin	99.7% specificity	Cross-validation
Al-Qirshi et al. (2024) [14]	Random forest	LUAD vs. LUSC	97.0%	Not reported
Cai et al. (2022) [15]	Deep learning	Cancer detection	AUC improved	Multi-cohort
This study	DNN	LUAD vs. LUSC	96.9%	Multi-cohort (88.9%)

## Data Availability

The DNA methylation datasets analyzed in this study are publicly available. The TCGA-LUAD and TCGA-LUSC datasets can be accessed through the Genomic Data Commons (GDC) portal (https://portal.gdc.cancer.gov/). The external validation datasets GSE39279 and GSE56044 are available from the Gene Expression Omnibus (GEO) repository (https://www.ncbi.nlm.nih.gov/geo/).
